# Integrated smart dust monitoring and prediction system for surface mine sites using IoT and machine learning techniques

**DOI:** 10.1038/s41598-024-58021-x

**Published:** 2024-03-30

**Authors:** Abhishek Kumar Tripathi, Mangalpady Aruna, Satyajeet Parida, Durgesh Nandan, P. V. Elumalai, E. Prakash, Joshua Stephen Chellakumar Isaac JoshuaRamesh Lalvani, Koppula Srinivas Rao

**Affiliations:** 1 Department of Mining Engineering, Aditya Engineering College, Surampalem, Andhra Pradesh 53347 India; 2 Department of Mining Engineering, National Institute of Technology Karnataka, Surathkal, 575025 India; 3 School of Computer Science & Artificial Intelligence, SR University, Warangal, Telangana 506004 India; 4 Department of Mechanical Engineering, Aditya Engineering College, Surampalem, India; 5 Metharath University, Bang Toei, 12160 Thailand; 6 Department of Mechtronics Engineering, Rajalaskhmi Engineering College, Mevalurkuppam, India; 7 Faculty of Mechanical Engineering, Arba Minch Institute of Technology, Arba Minch University, Arba Minch, Ethiopia; 8 Department of Computer Science and Engineering, MLR Institute of Technology, Hyderabad, Telangana India

**Keywords:** Mining industry, Miner's safety, IoT technology, Machine learning models, Dust pollutants, Environmental sciences, Energy science and technology, Engineering

## Abstract

The mining industry confronts significant challenges in mitigating airborne particulate matter (PM) pollution, necessitating innovative approaches for effective monitoring and prediction. This research focuses on the design and development of an Internet of Things (IoT)-based real-time monitoring system tailored for PM pollutants in surface mines, specifically PM 1.0, PM 2.5, PM 4.0, and PM 10.0. The novelty of this work lies in the integration of IoT technology for real-time measurement and the application of machine learning (ML) techniques for accurate prediction based on recorded dust pollutants data. The study's findings indicate that PM 1.0 pollutants exhibited the highest concentration in the atmosphere of the ball clay surface mine sites, with the stockyard site registering the maximum levels of PM pollutants (28.45 µg/m^3^, 27.89 µg/m^3^, 26.17 µg/m^3^, and 27.24 µg/m^3^, respectively) due to the dry nature of clay materials. Additionally, the research establishes four ML models—Decision Tree (DT), Gradient Boosting Regression (GBR), Random Forest (RF), and Linear Regression (LR)—for predicting PM pollutant concentrations. Notably, Random Forest demonstrates superior performance with the lowest Mean Absolute Error (MAE) and Root Mean Squared Error (RMSE) at 1.079 and 1.497, respectively. This comprehensive solution, combining IoT-based monitoring and ML-based prediction, contributes to sustainable mining practices, safeguarding worker well-being, and preserving the environment.

## Introduction

The mining industry, acknowledged as a hazardous sector, confronts substantial challenges, with worker safety as a paramount concern. Surface mining operations, integral to resource extraction, inevitably generate mineral dust, adversely affecting air quality within the mining environment^1,2^. This dust, a form of airborne particulate matter (PM), arises from diverse sources, including wind erosion, agricultural activities, and various anthropogenic processes^3^.

Inhalation of PM pollutants, especially mineral dust, poses severe health risks, leading to lung diseases like Pneumoconiosis^4^. This group of diseases, notably including silicosis, asbestosis, and coal worker pneumoconiosis (CWP), has debilitating consequences for miners' respiratory health^5^. Chronic exposure to PM dust in mining environments, categorized by size (PM 1.0, PM 2.5, PM 4.0, and PM 10.0), can result in diverse health hazards, from lung scarring to pulmonary diseases like cancer and bronchitis^6–8^.

The health impact on miners not only degrades their well-being but also places immense pressure on the industry^9^. Therefore, a safety-focused approach becomes imperative to mitigate occupational health diseases, enhance productivity, and lower costs. In this context, monitoring PM at mining sites is crucial. Traditional techniques, such as personal dust sampling equipment and gravimetric dust sampling, face limitations in measuring particles smaller than 2.5 µ and lack real-time capabilities. Moreover, the existing monitoring system not only fails to monitor smaller particles in mining conditions but also cannot provide accurate information about the dust cloud in the mining environment. Accurate tracking of the dust cloud condition would enable the control of mineworkers' exposure. Recognizing valuable insights from recent studies, including the work conducted by Kruzhilko et al.^10^, which scientifically substantiates the correction of work shift structures for granite quarry workers, it becomes apparent that not only monitoring but also strategic adjustments in work schedules may play a crucial role in minimizing workers' exposure to hazardous conditions. The investigation undertaken by the authors delves into the development of a real-time monitoring system while also considering the potential benefits of managing work schedules to reduce the time workers spend in dusty areas.

The specific objectives of this work involve the development of a real-time monitoring system for particulate matter (PM) pollutants, specifically PM1.0, PM2.5, PM4.0, and PM10.0, utilizing Internet of Things (IoT) technology. The aim is to effectively monitor PM dust pollution in surface mine sites, with a focus on PM 1.0, in real time. This will improves miners' health by safeguarding them against PM dust pollution by continuously and accurately tracking its concentration. The application of Internet of Things (IoT) technology in mining industry provides the efficient way to monitor the PM dust concentration in real time which detects the early pollution level in surface mine sites. Additionally, the research establishes four machine-learning models (Linear Regression, Gradient Boosting Regression, Decision Tree, and Random Forest) to predict PM pollutant concentrations. The study evaluates the performance of these models and identifies Random Forest as the most effective, with low Mean Absolute Error (MAE) and Root Mean Squared Error (RMSE). The integration of IoT technology and machine learning aims to provide valuable insights into assessing and managing air pollution levels in surface mines, contributing to sustainable mining practices and the protection of human health and the environment^11^.

After establishing the specific objectives, the PM pollutant measuring device prototype was developed, comprising a Sensirion SPS30 sensor for detecting airborne dust concentration, a radio transceiver, a microcontroller, and a display unit. The sensor sends the detected information to the signal conditioning and computational unit, which then transmits the data to the radio transceiver for establishing Bluetooth or wireless communication. Subsequently, the IoT-based PM monitoring technique was implemented in a ball clay open-pit quarry in West Godavari District, Andhra Pradesh, to achieve the outlined objectives. The measurements of weather parameters along with PM dust pollution were conducted from morning 8:00 a.m. to evening 8:30 p.m. during 26th April 2022 to 30th April 2022, at 15-min intervals.

The paper is structured into nine sections, covering the introduction, literature review, system development, field study, results and discussion, Prediction of Air Pollution Levels using various machine learning approach which is followed by model evaluation, practical Applications and Future Research Prospects and conclusions.

## Literature review

In mining operations, the generation of dust is a frequent phenomenon, leading to the presence of airborne dust suspended in the mine atmosphere. This airborne dust primarily comprises mineral particles and, in the presence of moisture, gives rise to particulate matter, which consists of a complex mixture of solid and liquid components. The size of these particles ranges from 10 µ to 2.5 µ, rendering them invisible to the naked eye. Inhalation of such particles can pose significant health hazards to workers, especially upon chronic exposure. Particulate matter is composed of a combination of organic and inorganic particles, including dust, pollen, soot, smoke, and liquid droplets, making it extremely hazardous to human respiratory health. Thus, monitoring the levels of particulate matter in mining sites is of utmost importance for ensuring the safety and well-being of workers. This monitoring plays a vital role in the prevention and prediction of health hazards associated with inhalation^12^.

### Understanding particulate matter in mining environments: sources, characteristics, and health impact

Understanding particulate matter (PM) in mining environments is essential for recognizing its sources, characteristics, and the potential impact it poses in the mining context. PM originates from both natural and anthropogenic sources, encompassing sea salt, pollen, volcanic eruptions, airborne dust, and various industrial activities. Among industrial operations, mining significantly contributes to PM emissions due to processes such as drilling, blasting, transportation, and handling of materials. Drilling operations generate suspended airborne dust particles, while blasting releases particles and gas emissions, including NOx, which can pose health risks^13^. Additionally, open-pit coal mining contributes to elevated PM levels, facilitated by wind-driven dispersion of coal dust, necessitating the implementation of effective mitigation strategies to safeguard human health and the environment^14^.

Based on their formation mechanisms, PM is classified into various types, including dust, smoke, fumes, fly ash, mist, and spray (see Table 1). These different PM types exhibit distinct size ranges, with fine and ultrafine particles capable of reaching the alveoli in the respiratory system, while PM10-sized particles primarily settle in the upper airways. The percentage of inhaled airborne particles that enter the respiratory tract is represented by total inhalable dust^15^. Other measures, such as thoracic and respirable dust, refer to particles that pass through the larynx into the thoracic cavity and reach the gas exchange region of the lungs, respectively. Hazardous dusts can also chemically interact with the respiratory system, allowing toxic substances like lead and arsenic to pass through alveolar walls into the bloodstream^16^. A comprehensive understanding of these PM classifications is crucial for assessing their impact on human health.

**Table 1:** Size ranges of pm types.

Sl. no.	PM classification	Size ranges (µm)
1	Fumes	0.03 to0.3
2	Smoke	0.50 to 1.0
3	Mist	≤ 10
4	Fly ash	1.0 to 1000
5	Spray	10 to 1000
6	Dust	1.0 to 10,000

### Importance and methods of monitoring particulate matter in various environments

Exposure to particulate matter (PM) poses significant health risks to miners, as they inhale ambient air in their workplace. PM's mineralogical composition can lead to severe health issues, such as asbestosis and silicosis^3^. Effective monitoring of PM is crucial not only for environmental permits and planning but also for safeguarding miners' health. However, current monitoring systems in mining areas encounter limitations, necessitating the implementation of fast and accurate air monitoring systems. Inadequate monitoring of PM dust concentration (ranging from PM 2.5 µ to PM 10 µ) can lead to worker exposure and various health complications, including respiratory problems, lung diseases, breathing difficulties, non-fatal heart attacks, and cardiac arrhythmias. Therefore, comprehensive and precise monitoring systems are essential for ensuring the well-being of miners^17,18^.

Monitoring particulate matter (PM) in mining sites involves collecting air quality data while considering wind direction. This monitoring can be divided into three parts: (1) monitoring the mine atmosphere away from equipment operations but within the site, (2) monitoring PM dust at operating sites, including drilling, blasting, loading, transportation, and facilities, and (3) monitoring PM dust outside the mining area^19^.

### IoT-based monitoring systems for particulate matter: a review of past research work

In mining operations, various dust-forming activities occur at different locations, necessitating the monitoring of particulate matter (PM) concentrations at multiple sites. The rapid advancement of Internet of Things (IoT) technology has led to the development of IoT-based PM monitoring systems, which serve as a promising alternative to traditional monitoring methods^20^. Conventional monitoring systems often require significant human intervention, are time-consuming, and may result in manual errors, emphasizing the need for improved monitoring solutions. IoT-based PM monitoring systems collect data through measurement devices (sensors) and transmit it via the network, making them more efficient and reliable. These systems are designed to enable mine operators to promptly inspect dust-causing sites and implement necessary preventive measures. To be effective, these systems should be easy to install at multiple sites and exhibit sufficient endurance, considering that the main dust-generating areas may change over time, and workers are exposed to harsh outdoor conditions during mining operations. This study investigates the performance of IoT measurement devices and the network in existing operations, including an open-pit mine site.

A multitude of studies has explored the application of the Internet of Things (IoT) in tracking traffic flow and monitoring air quality. For instance, a study in 2022 introduced an inexpensive IoT-based system for tracking traffic flow and determining the air quality index (AQI)^21^. This study utilized machine learning methods, which eliminated the need for complex calibration, allowing the measurement of pollutant gases and accurate determination of AQI. Similarly, another study in 2020 demonstrated an IoT-based indoor air quality monitoring platform, storing data in the cloud and providing resources for further indoor air quality studies^22^.

In line with this, researchers in 2020 developed an IoT system for monitoring air quality, capable of monitoring local air quality and providing data for user analysis via an integrated buzzer^23^. Additionally, another study in 2020 discussed the use of IoT in the mining field, highlighting how IoT serves as a wireless network for collecting information from electronic devices and sensors^24^.

Over the past decade, advances in wireless sensor networks (WSN), radio frequency identification (RFID), and cloud computing have facilitated the integration of the Internet of Things (IoT) in harsh work environments like mining^25^. This integration has significantly improved the accuracy, efficiency, cost-effectiveness, and real-time capabilities of the monitoring process. Notably, these advancements have enabled automatic event detection, control, and remote data exchange, making monitoring feasible in otherwise inaccessible locations. Several successful implementations of WSN-based monitoring systems have been reported, such as early detection of fires in coal mines and detection of toxic mine gases in the environment. Furthermore, IoT technology has enabled the accurate measurement of particulate matter within a short time. Given that time and cost are crucial factors in managing these projects, this work aims to develop a low-cost IoT-based PM monitoring device capable of monitoring pollutants of less than 2.5 µ. By utilising these technologies, mining operations can be made safer and more efficient, while simultaneously reducing costs and environmental impacts^26^.

### Predicting particulate matter: an application of AI and ML

Numerous studies have proposed various machine learning algorithms for the prediction of airborne particulate matter. Li et al. introduced a real-time prediction approach based on weighted extreme learning machine (WELM) and adaptive neuro-fuzzy inference system (ANFIS)^27^. Choubin et al. developed machine learning models, including Random Forest (RF), Bagged Classification and Regression Trees (Bagged CART), and Mixture Discriminant Analysis (MDA), for forecasting PM10-induced risk^28^. Rutherford et al. utilized excitation-emission matrix (EEM) fluorescence spectroscopy and a machine learning algorithm to localize PM sources^29^.

In the context of PM2.5 prediction, Just et al. proposed a new strategy using machine learning techniques^30^. Yang et al. put forward hybrid models by combining different deep learning approaches^31^. Stirnberg et al. developed a method integrating satellite-based Aerosol Optical Depth (AOD) with meteorological and land use factors for predicting PM10 concentrations^32^. Additionally, Gilik et al. constructed a supervised model for air pollution prediction using sensor data and explored model transferability between cities^33^.These comprehensive studies collectively demonstrate the potential and effectiveness of machine learning in air pollution prediction, providing valuable insights for future research and applications in this field.

In the context of this literature review, the section prominently highlights the novelty and scientific contribution of the current research work—an IoT-based monitoring and ML powered dust prediction system. The proposed system not only offers real-time monitoring of various PM particle sizes, including PM1.0, PM2.5, PM4.0, and PM10.0, but also integrates a efficient prediction model to ensure precise and accurate PM measurements. With hardware integration and robust software protocols, the system addresses the limitations of traditional monitoring techniques, facilitating efficient and comprehensive monitoring of PM dust concentration in mining environments. This research work aims to significantly contribute for improving mine air quality by effectively monitored and prediction of PM dust pollution in surface mine sites by utilising the cutting edge technology like IoT and ML. The proposed IoT-based Dust Monitoring System stands as a novel and practical solution that advances the field of air quality monitoring and holds promising potential for widespread implementation in mining and beyond.

## IoT-based dust monitoring system: development and implementation

This section delves into the characteristics and system architecture of the proposed solution, designed to offer real-time monitoring of PM concentrations, including PM1.0, PM2.5, PM4.0, and PM10.0, within surface mine sites. The integration of advanced hardware, data transmission protocols, and machine learning techniques provides a comprehensive and efficient approach to monitor and predict airborne PM levels. By addressing the limitations of traditional monitoring methods, this system aims to significantly enhance mine air quality assessment, reduce miners' exposure to harmful air, and mitigate PM pollution, by efficiently predicting it in real working environment. In the following subsections, we detail the characteristics of the IoT monitoring system and present its system architecture, elucidating the technical aspects and the novel contributions of this research.

### Characterstics of IoT monitoring system

IoT monitoring systems are integral to the successful implementation of real-time particulate matter (PM) monitoring in mining environments. Typically comprising a transmitter and a receiver powered by a battery, these systems play a pivotal role in gathering and transmitting crucial data. At the heart of any IoT project lies the sensing device, responsible for measuring dynamic physical quantities and converting them into proportional electrical signals. Among the commonly used sensors for detecting dust, smoke, and particles is the Dust Sensor, capable of detecting tiny particles larger than 0.8, including cigarette smoke.

The particulate matter (PM) sensor used in this study, with the model number Sensirion SPS30, was manufactured in China. It measured different size concentrations, ranging from PM10, PM4.0, PM2.5, to PM1.0. This sensor employs laser scattering technology to detect particulate matter and is renowned for its accuracy, reliability, and extended lifespan. With an operating temperature considered as high as 63 °C, the SPS30 demonstrates long-term stability, making it well-suited for mining environments. The electrical characteristics of the Sensirion SPS30 Sensor, used for detecting particulate matter and monitoring air quality in surface mines, are detailed in Table 2. To ensure optimal performance, it is recommended to operate the SPS30 sensor within the normal temperature range of 10–40 °C and a humidity range of 20–80% RH.

**Table 2:** Electrical characteristic of SPS30 sensirion dust sensor.

Parameter	Notation	Value	Units
Supply voltage	V_s_	4.5–5.5	volt
Idle current	I_d_	< 8	milliampere
Average supply current	I_avgs_	60	milliampere
Maximum supply current	I_maxs_	80	milliampere

For achieving seamless connectivity and enabling data transmission, this study utilizes a low-cost, low-power microcontroller chip with an integrated Wi-Fi module. The model employed is ESP-32, manufactured in China. This module supports both Bluetooth and Wi-Fi connectivity while also supplying the required power for the sensor's operation. The monitoring system is equipped with a display panel to present particulate matter presence results, facilitating real-time access to information.

The Sensirion SPS30 sensor operates in three different modes: idle, measurement, and sleep mode. In idle mode, the fan and laser consume the most power during the initialization phase when the sensor module is turned on and ready to detect fine dust and process commands. The measurement mode is continuously monitored, where all electronic components consume maximum power, and data are measured every second. The system efficiently transitions to sleep mode from the measurement mode. During this transition, all internal components reduce power consumption, the microcontroller goes into sleep mode, and the UART/I2C interface is disabled, conserving energy and optimizing operational efficiency.

The IoT-based Dust Monitoring System exhibits cutting-edge technology, precision, and adaptability, making it a powerful tool for safeguarding miners' health and enhancing mine air quality assessment. Through the utilization of state-of-the-art sensors and communication protocols, this system represents a novel contribution that addresses the limitations of traditional monitoring techniques in surface mines air pollution measurement and prediction. Its real-time monitoring capabilities for dust concentration in the mining environment pave the way for proactive measures in reducing the adverse effects on miners' health. If the recorded dust concentration exceeds permissible limits, the system can trigger the implementation of suitable dust suppression mechanisms. Examples of these mechanisms include Water sprinklers and spray bars, Water trucks, Fog cannons, and Chemical dust suppressants. These measures play a crucial role in mitigating potential health risks associated with elevated dust levels in surface mines. This comprehensive approach not only advances the field of particulate matter monitoring but also establishes the IoT-based Dust Monitoring System as an indispensable asset in promoting a safer and healthier working environment for surface miner.

### System architecture

**Figure 1:**
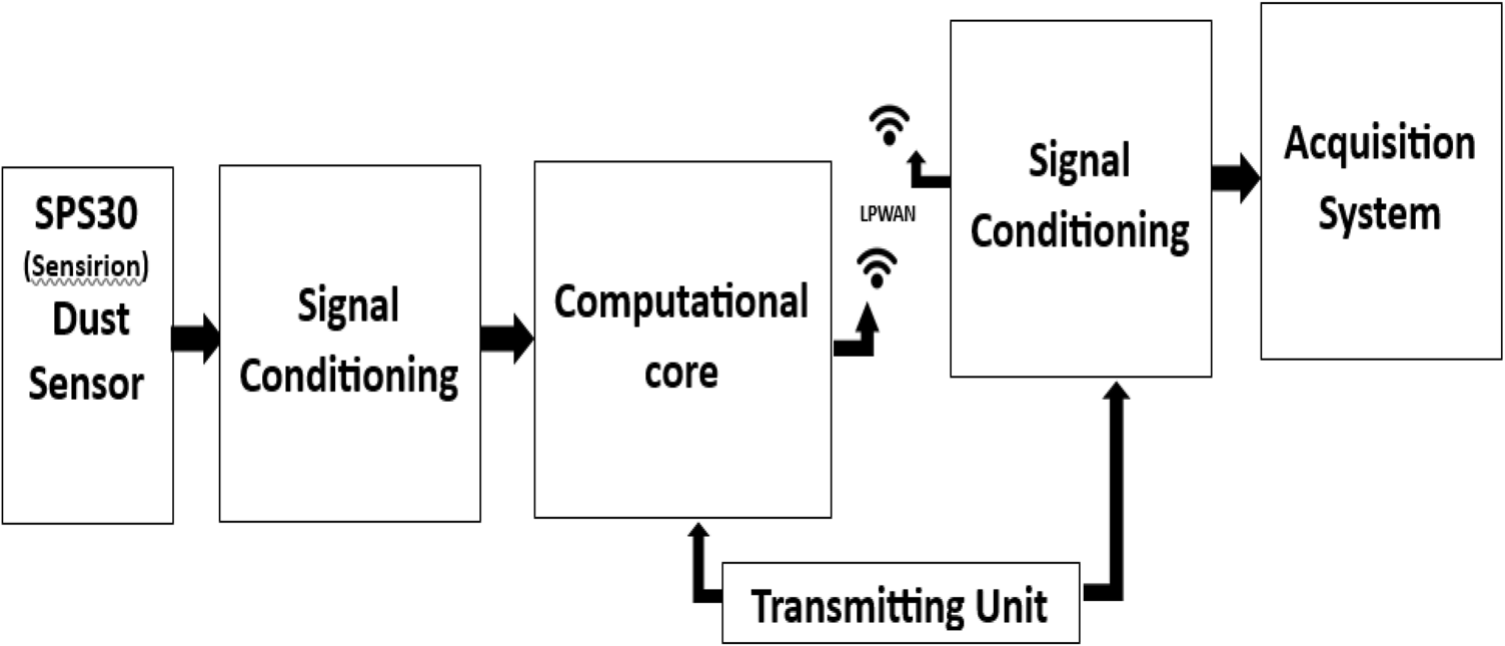
Functional architecture of sensing node's transmitter and receiver in an IoT based monitoring system.

The system architecture of the proposed IoT-based Dust Monitoring System is a pivotal aspect that defines its structure, behavior, and essential components. This conceptual model provides a detailed, systematic, and diagrammatic representation of how the system operates, with a specific focus on monitoring particulate matter in surface mine sites. Figure 1 illustrates the functional architecture, encompassing the IoT sensor, transmitter, and receiver units, forming the backbone of the monitoring system.

The monitoring system comprises key components, including the highly efficient Sensirion SPS30 sensor, breadboard, OLED display, ESP32 (30 pins), NodeMcu baseboard, and female-to-female cable jumper. These devices are skillfully interconnected on a robust wooden board, ensuring stability and portability. The system architecture, depicted in Fig. 3, initiates fine dust monitoring by initializing the ESP32 chip, responsible for providing Wi-Fi and dual-mode Bluetooth connectivity. The ESP32 chip operates autonomously or as a subordinate device to a host MCU, effectively reducing the communication stack overhead. At the core of the system lies the Sensirion SPS30 sensor, adept at detecting and measuring PM1.0, PM2.5, PM4.0, and PM10.0 particles. The sensor converts the detected readings into electrical signals, efficiently transmitted to the ESP32 and NodeMCU within the transmission unit.

In the subsequent stages, the ESP32 efficiently sends the data to the display unit, comprising cloud storage, an OLED display, and serial monitoring. This integration enables seamless visualization of the collected data, allowing for real-time analysis and interpretation of particulate matter concentrations. The cloud storage component ensures data retention for further analysis and historical reference.

The proposed system architecture demonstrates a highly functional and sophisticated design, efficiently capturing and processing data on particulate matter concentrations. By integrating cutting-edge components and communication protocols, the system guarantees reliable and accurate monitoring, paving the way for improved mine air quality assessment and timely preventive measures. The versatility and robustness of this architecture present a novel and practical solution to address the limitations of traditional monitoring techniques, offering a significant advancement in monitoring airborne particulate matter within surface mine environments.

## Methodology: implementation of IoT-based dust monitoring system in field study

**Figure 2:**
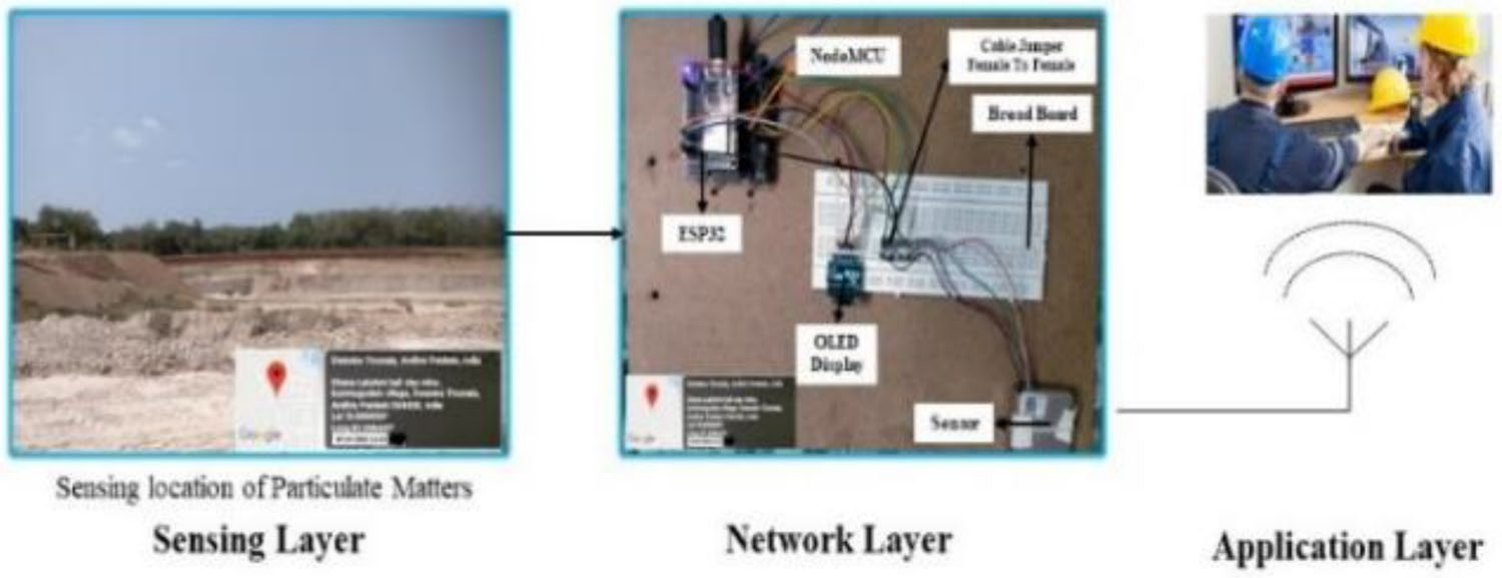
IoT based particulate matter measurement system in surface mines.

The field study was conducted over a span of 5 days, from April 26, 2022, to April 30, 2022, at the ball clay surface mine sites located in Kommugudem village, Andhra Pradesh, India situated at latitude of 16° 57′ 22.5288″ and longitude of 81° 15′ 21.6468″. Figure 2 represents the proposed IoT based PM dust monitoring technique. The primary objective of this study was to monitor, in real time, the concentration of PMs in the atmosphere of mining environment using IoT based monitoring system. The collected data through IoT system was further used to predict the air pollution level in mines by using various sophisticated machine learning (ML) techniques, such as Linear Regression (LR), Gradient Boosting Regression (GBR), Decision Tree (DT), and Random Forest (RF). This ML techniques were efficiently predicts the concentration PM 1.0, PM 2.5, PM 4.0, and PM 10.0 levels at the loading and stockyard point of ball clay surface mine. The efficacy of developed ML techniques were compared using performance metrics namely mean absolute error (MAE) and root mean square error (RMSE).

Figure 2 showcases the setup of the IoT-based monitoring system at the mine site, emphasizing particulate matter monitoring at both the loading point and the stockyard. Previous literature reports higher PM concentrations of ball clay at its stockyard than at the loading point. To validate this observation, a real-time IoT-based dust monitoring device was deployed at two distinct locations: the loading point and stockyard in surface ball clay mine. The device provides a real-time response of measured dust concentrations in terms of PM1.0, PM2.5, PM4.0, and PM10.0. The obtained findings were analyzed based on previously reported work. Over a 5-day field measurement period, monitoring was conducted for 2 days near the loading point and 3 days near the stockyard of the selected ball clay mines.

Throughout the field study, the Sensirion SPS30 sensor played a crucial role in measuring particulate matter of varying sizes, including PM10, PM4.0, PM2.5, and PM1.0. Readings were recorded every 15 min between 8:00 am and 8:30 pm on each study day, resulting in a total of 50 readings for each particulate matter size. By analyzing this data, the system provided real-time insights into particulate matter concentrations, enabling the identification of peak and low points for further analysis and necessary preventive measures against particulate matter pollution.

**Figure 3:**
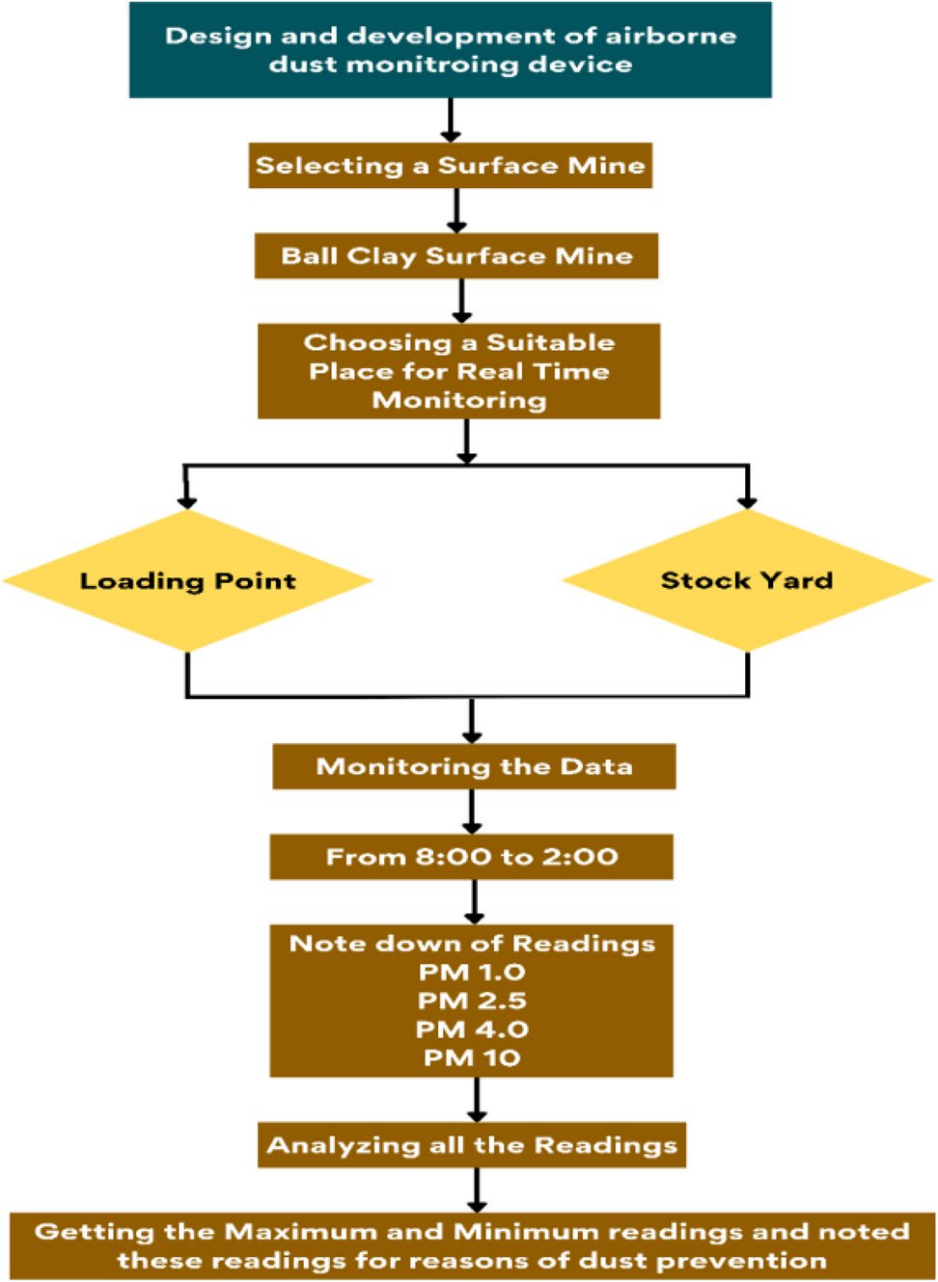
Flow chart of field study.

Figure 3 illustrates the detailed flow chart of the field study, outlining the sequence of activities and data collection process. The recorded maximum and minimum particulate matter values served as crucial parameters in determining the appropriate measures to mitigate particulate matter pollution effectively.

To address the issue of dust concentration and ensure worker safety, a dust suppression system was evaluated during the field study. This system involves the addition of liquid, such as water or surface-attracting agents, to airborne dust particles. Atomizing nozzles are strategically placed to spray a fine mist of water through dust cannons onto the airborne particles, effectively suppressing up to 95% of mobile dust generated during various mining operations, including drilling, dumping, crushing, and loading into trucks. Additionally, water sprinklers were employed on transport routes to further reduce dust levels. The use of dust suppression techniques was evaluated for its effectiveness in improving air quality and reducing health hazards posed by particulate matter exposure in mining environments.

The field study provided valuable data and insights into the dynamic nature of particulate matter concentrations in the mining environment. By leveraging the IoT-based monitoring system and assessing the effectiveness of dust suppression methods, this study contributes to enhancing safety standards and environmental protection in surface mine sites. The results obtained offer essential guidance for future mining operations to mitigate the adverse effects of particulate matter pollution and prioritize the well-being of miners and surrounding communities.

## Results and discussion-field study

**Figure 4:**
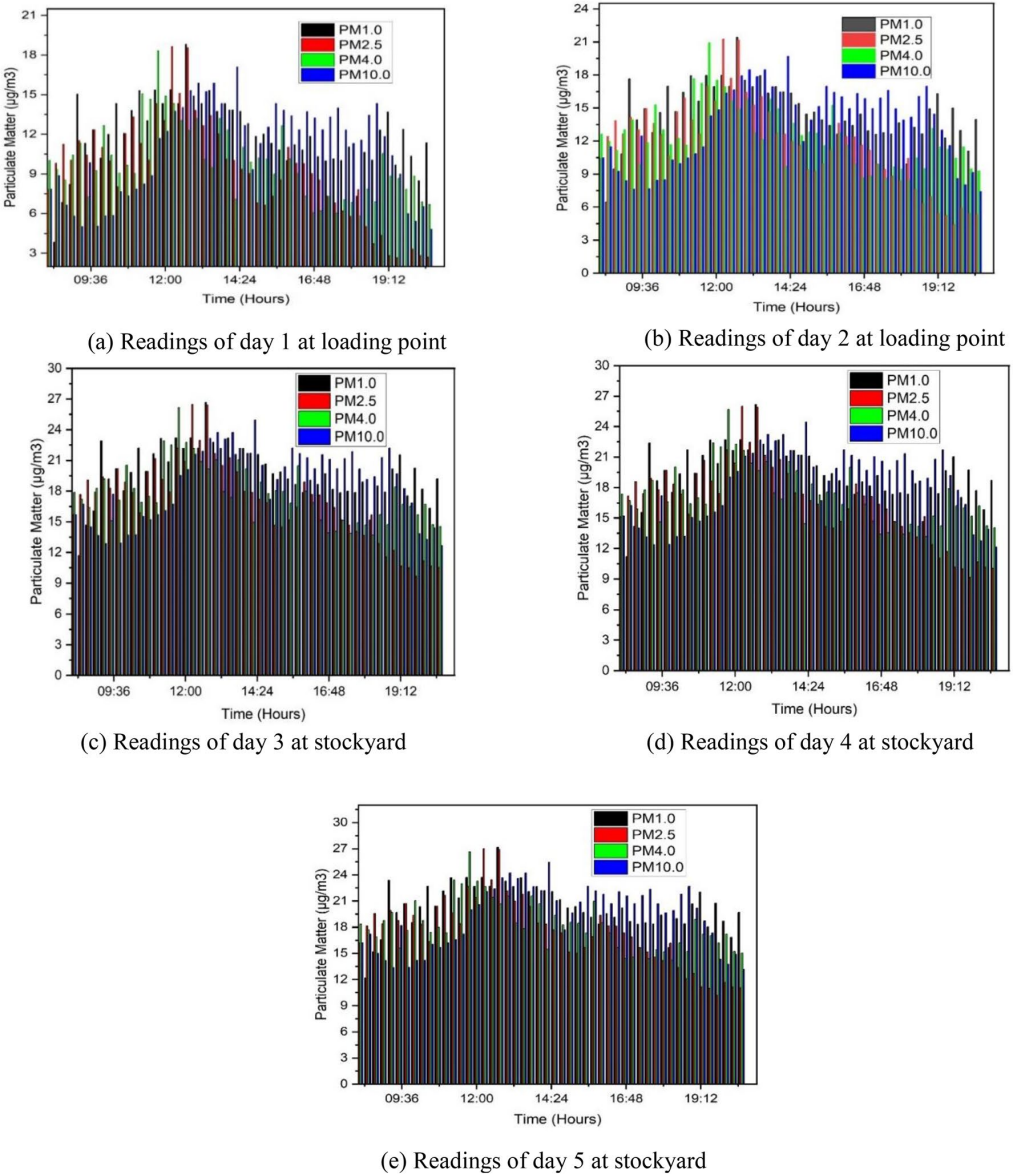
Combined particulate matter levels recorded at loading site and storage yard for PM 1.0, PM 2.5, PM 4.0, and PM 10.0.

The results of the 5-day IoT-based field study for particulate monitoring are consolidated in Fig. 4. As depicted, PM pollutant readings were monitored at two distinct locations: the loading point and stockyard. Readings for days 1 and 2, associated with the loading point, are presented in parts (a) and (b) of Fig. 4. Conversely, PM pollutant readings corresponding to days 3, 4, and 5 for the stockyard are depicted in parts (c), (d), and (e) of Fig. 4. The combined particulate matter levels recorded at the loading site and stockyard for PM 1.0, PM 2.5, PM 4.0, and PM 10.0 are represented by black, red, green, and blue colors, respectively.

The recorded maximum and minimum values of PM1.0, PM2.5, PM4.0, and PM10.0 for all 5 days are summarized in Table 3. A thorough examination of the daily readings reveals distinct patterns in the concentrations of different particulate matter sizes. It was observed that PM1.0 exhibits the maximum concentration among the four particulate matters, while PM2.5 shows the minimum concentration. PM1.0 reached a maximum concentration of 18.23 µg/m^3^ at the loading point, while PM2.5, PM4.0, and PM10.0 exhibited concentrations of 18.17 µg/m^3^, 17.99 µg/m^3^, and 16.95 µg/m^3^, respectively, for day 1 at the loading point. Similarly, on the second day, particulate matter of PM1.0, PM2.5, PM4.0, and PM10.0 reached maximum concentrations of 21.42 µg/m^3^, 20.85 µg/m^3^, 20.93 µg/m^3^, and 19.70 µg/m^3^, respectively.

**Table 3:** Maximum and minimum values of PM pollutants of all the 5 day.

Particulate matter	Day									
	Loading point				Stock yard					
	Day 1		Day 2		Day 3		Day 4		Day 5	
	PM_max_ (µg/m^3^_)_	PM_min_ (µg/m^3^_)_	PM_max_ (µg/m^3^_)_	PM_min_ (µg/m^3^_)_	PM_max_ (µg/m^3^_)_	PM_min_ (µg/m^3^_)_	PM_max_ (µg/m^3^_)_	PM_min_ (µg/m^3^_)_	PM_max_ (µg/m^3^_)_	PM_min_ (µg/m^3^_)_
PM1.0	18.23	3.86	21.42	6.46	27.10	10.7	26.83	11.62	28.45	12.20
PM2.5	18.17	1.83	20.85	4.43	25.96	9.67	25.30	9.17	27.89	10.17
PM4.0	17.99	5.85	20.93	8.45	26.07	13.69	27.63	13.19	26.17	14.19
PM10.0	16.95	4.82	19.70	7.42	24.04	11.76	25.19	12.85	27.24	13.16

For the stockyard, PM1.0 consistently exhibited the highest concentrations, with maximum values of 27.10 µg/m^3^, 26.83 µg/m^3^, and 28.45 µg/m^3^ on days 3, 4, and 5, respectively. On day 3, the maximum values of PM2.5, PM4.0, and PM10.0 were 25.96 µg/m^3^, 26.07 µg/m^3^, and 24.04 µg/m^3^, respectively. Likewise, the maximum concentrations of PM2.5, PM4.0, and PM10.0 for day 4 were 25.30 µg/m^3^, 27.63 µg/m^3^, and 25.19 µg/m^3^, respectively. The maximum concentrations of PM2.5, PM4.0, and PM10.0 for day 5 were 27.89 µg/m^3^, 26.17 µg/m^3^, and 27.24 µg/m^3^, respectively.

During the study, higher dust concentrations were observed in the stockyard compared to the loading point. This phenomenon is attributed to the typical nature of Ball clay minerals, abundant in the earth's crust, which are known to release minimal particulate matter during loading. However, once the ball clay material is transported to the stockyard, the effects of air movement result in the release of fine particulate matter into the atmosphere. Consequently, more significant dust concentrations can be observed at the stockyard compared to the loading point, a trend also observed in previous studies. This substantiates the effectiveness of field validation for the developed IoT-based PM dust monitoring device in the surface mine environment.

This detailed analysis underscores the significance of PM1.0 in the atmosphere of the investigated surface mines, suggesting potential health risks to miners. The consistently higher concentrations at the stockyard, particularly for PM1.0, can be attributed to the drier nature of clay materials in this location, leading to increased air pollution. The highest levels of PM pollutants at both the stockyard and loading point were consistently recorded between 11:45 a.m. and 2:00 p.m. This observation reinforces the understanding that dry clay materials are more prone to stirring up air pollution compared to wet clay, typically encountered during loading. This finding indicates that the peak interval for PM dust pollution occurs from 11:45 a.m. to 2:00 p.m. in ball clay surface mines. Therefore, during this period, extra care in implementing dust control measures is required in ball clay surface mines to effectively control the intensity of PM dust pollution.

While acknowledging that the developed IoT-based real-time monitoring system serves as a demonstration of the current situation without directly reducing danger, it is emphasized that its significance lies in enabling proactive dust mitigation measures. The real-time monitoring of dust concentration facilitates timely decision-making to adopt preventive measures before reaching levels that could pose harm to miners. This approach enhances overall safety and health by providing crucial data for informed and swift actions in implementing proactive dust suppression or containment measures. These findings emphasize the importance of implementing effective preventive measures to mitigate particulate matter pollution and ensure the safety and well-being of miners and the surrounding environment.

## Prediction of air pollution levels: data description and modeling

To predict PM 1.0, PM 2.5, PM 4.0, and PM 10.0 concentrations at the ball clay surface mine sites, four distinct machine learning models, namely, Linear Regression (LR), Gradient Boosting Regression (GBR), Decision Tree (DT) and Random Forest (RF), and, were employed. These models were trained on a dataset obtained during a 5-day field study, encompassing 250 data points. The dataset includes essential information such as time of measurment, weather conditions, wind direction, and other pertinent factors, which serve as input features for the models. The objective is to accurately estimate particulate matter concentrations at different sizes, enabling an effective assessment of air pollution levels.

To develop the particulate matter prediction model, data from IoT sensors measuring PM levels and weather-related parameters, including solar radiation (W/m^2^), air temperature (°C), relative humidity (%), and wind speed (m/s), were recorded. The primary objective was to explore potential patterns and correlations within the dataset to enhance the accuracy of predicting particulate matter levels.

### Data description

The dataset used for training the prediction models comprises 250 data points collected during the 5-day field study conducted at the ball clay surface mine site. Table 4 provides a comprehensive overview of the collected data, encompassing crucial meteorological parameters and concentrations of particulate matter. The meteorological parameters include solar radiation (W/m^2^), air temperature (°C), relative humidity (%), and wind speed (m/s).

**Table 4:** Description of the data used for modelling.

Attribute	Solar radiation (W/m^2^)	Air temperature (°C)	Relative humidity (%)	Wind speed (m/s)	PM1.0 (µg/m^3^)	PM2.5 (µg/m^3^)	PM4.0 (µg/m^3^)	PM10.0 (µg/m^3^)
Count	250	250	250	250	250	250	250	250
Mean	518.57	30.25	63.27	4.07	17.09	14.43	15.08	16.10
Std	344.07	2.93	5.27	1.25	4.32	5.12	4.33	4.79
Min	0	21.88	6	0.90	3.86	1.83	5.85	4.82
25%	187.75	28.14	60	3.13	13.878	10.66	12	13.20
50%	589	30.96	60	4.07	17.89	14.66	15.24	16.04
75%	856.50	32.68	68	4.57	20.32	18.16	17.87	20.20
max	966	34.74	77	8.94	27.17	27	26.67	25.44

### Machine learning models

Four machine learning models were developed for predicting the concentrations of particulate matter at the ball clay open-pit mine. These models were chosen for their effectiveness in regression tasks and their ability to handle complex relationships between input features and output variables. Linear Regression (LR) takes a linear approach, modeling the relationship between the dependent variable and independent variables by fitting a linear equation. The Gradient Boosting Regression (GBR) model is an ensemble approach that sequentially builds multiple weak learners, each correcting the errors of its predecessor. The Decision Tree (DT) operates as a tree-like model, making decisions by partitioning the data into subsets based on input features. Random Forest (RF), another ensemble model, constructs numerous decision trees and merges their predictions to enhance accuracy and control overfitting.

**Figure 5:**
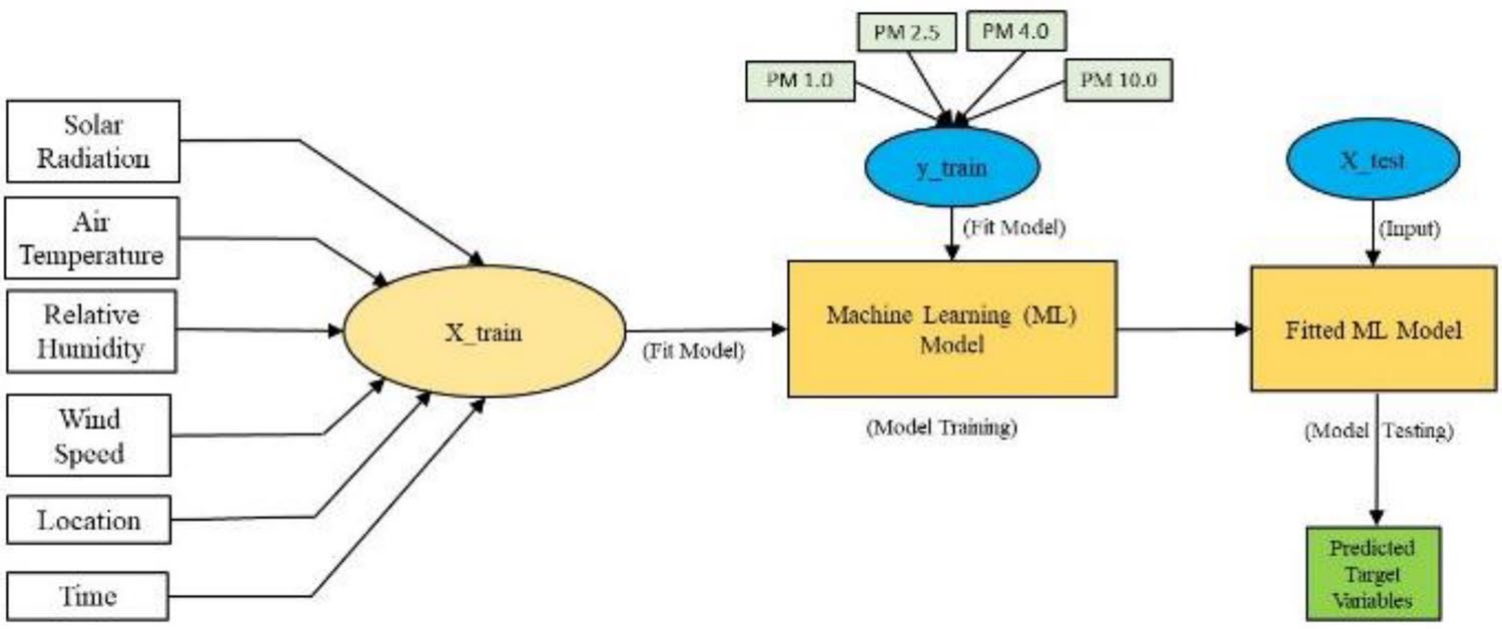
Workflow of the developed models.

The workflow of the models is depicted in Fig. 5, illustrating the process from input variables to predicting PM concentrations. The performance of each model was assessed using key metrics, including accuracy, root mean squared error (RMSE), and mean absolute error (MAE) values. The subsequent section will present the results, offering a comprehensive evaluation of the predictive capabilities of each machine learning approach in estimating air pollution levels at the ball clay surface mine site.

## Model evaluation and performance comparison

**Figure 6:**
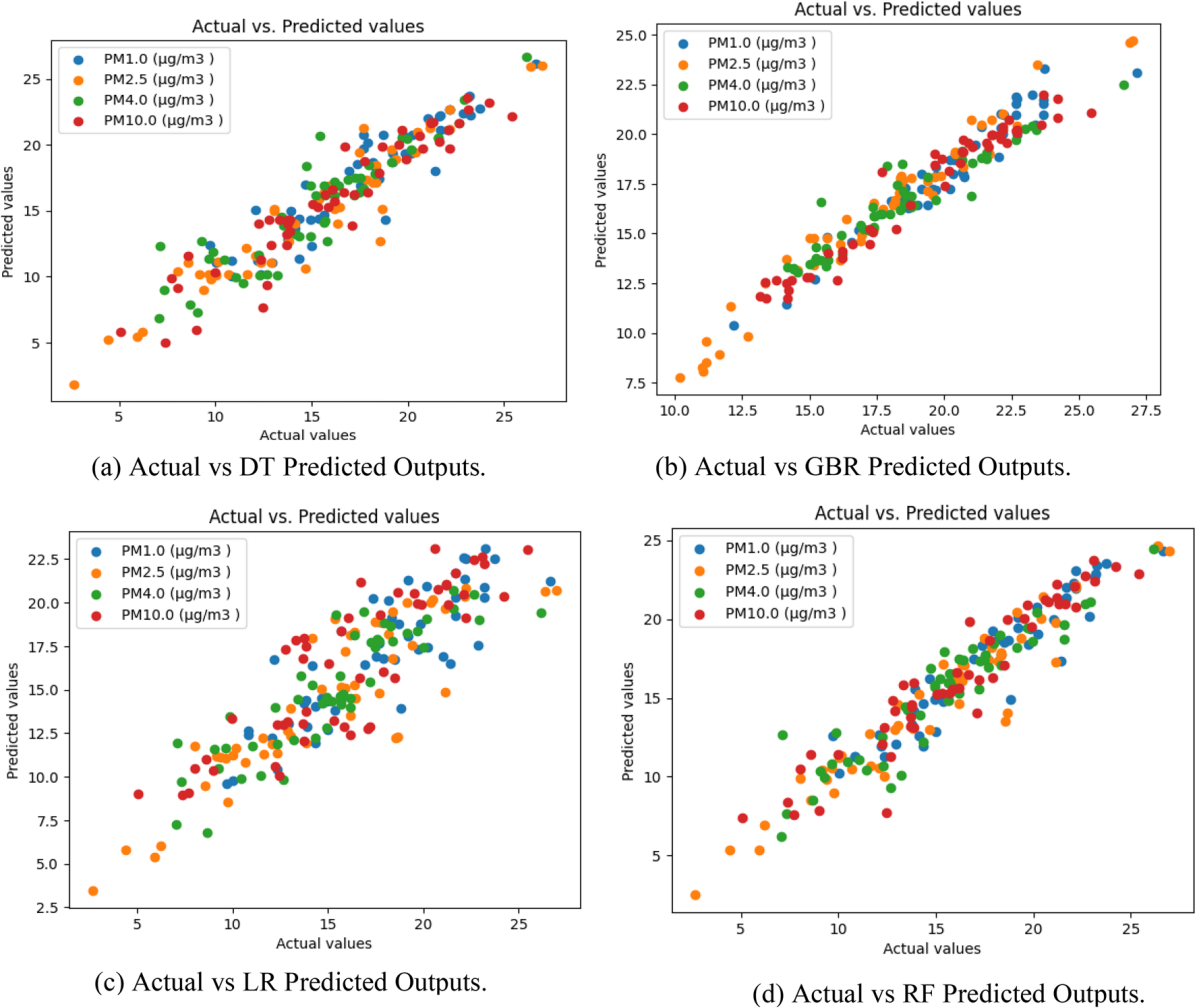
Representation of the correction between actual and predicted outputs.

In this section, the performance of four machine learning models—Decision Tree (DT), Gradient Boosting Regression (GBR), Random Forest (RF), and Linear Regression (LR)—developed for predicting particulate matter concentrations during a 5-day field study at the ball clay surface mine is comprehensively evaluated. The dataset, consisting of 250 data points, underwent a split into training (60%), testing (20%), and validation (20%) sets. The correlations graph between actual and predicted responses of the developed ML models is presented in Fig. 6.

Evaluation metrics, including Mean Absolute Error (MAE) and Root Mean Squared Error (RMSE), provide insights into the models' accuracy in predicting particulate matter dust concentrations. The Linear Regression (LR) model exhibits an MAE of 1.771 and an RMSE of 2.315, demonstrates suboptimal performance, displaying higher randomness in predictions, as depicted in Fig. 6. Further, the Gradient Boosting Regression (GBR) model achieves an MAE of 1.857 and an RMSE of 2.01, showcasing superior accuracy for PM 4.0 and PM 10.0 concentrations. Notably effective in the concentration range of 15 µg/m^3^ to 20 µg/m^3^, the GBR model outperforms in this specific range.

On the other hand, The Decision Tree (DT) model exhibits an MAE of 1.242 and an RMSE of 1.648, demonstrating good accuracy across all outputs. It particularly excels in predicting PM 1.0, PM 4.0, and PM 10.0 concentrations, with lower accuracy for concentrations below 15 µg/m^3^ but improvement above this threshold. The Random Forest (RF) model emerges as the top performer, achieving an MAE of 1.079 and an RMSE of 1.497 for all PM concentrations. Figure 6 illustrates its accurate predictions, especially within the 15 µg/m^3^ to 25 µg/m^3^ range, showcasing its effectiveness. The overall model evaluation and performance comparison highlight the superiority of the Random Forest (RF) model in accurately predicting PM 1.0, PM 2.5, PM 4.0, and PM 10.0 concentrations. The versatility of RF, combining multiple decision trees to handle complex relationships and reduce overfitting, contributes to its effectiveness in capturing patterns and interactions in real-world datasets.

## Practical applications and future research prospects

The IoT-based real-time monitoring system developed for surface mines holds promising practical applications and unveils intriguing avenues for future research. In practical terms, the system serves as a crucial tool for preemptive dust control, providing timely data that enables mine operators to activate targeted dust suppression measures, such as water sprinklers or chemical agents, before pollutant levels reach hazardous thresholds. Its integration with machine learning models facilitates not only real-time monitoring but also predictive analysis, allowing for proactive implementation of dust mitigation strategies. The autonomous actuation mechanism, currently a focal point of future research, aims to add a layer of sophistication to the system, enabling it to autonomously trigger specific dust control measures based on the detected pollutant levels.

Looking ahead, ongoing research could explore the integration of additional environmental parameters to enhance the predictive accuracy of the system. This may involve incorporating geological characteristics, meteorological conditions, and specific mining activities into the modeling framework. The deployment of the system across multiple mining sites could provide valuable insights into variations in pollutant concentrations across diverse environments. Moreover, research could delve into developing adaptive actuation algorithms, ensuring that the system dynamically adjusts dust mitigation measures in response to evolving conditions, thus optimizing its effectiveness. These avenues of exploration collectively highlight the transformative potential of the developed system in shaping the future of air quality management in surface mining, emphasizing the need for continued innovation and advancement in the field.

## Conclusions

The study emphasizes the vital role of real-time monitoring in addressing particulate matter (PM) pollution in surface mine sites and neighboring residential areas. Utilizing an IoT-based system, PM1.0, PM2.5, PM4.0, and PM10.0 concentrations were monitored at loading and stockyard sites, revealing elevated levels, especially at the stockyard site due to dry clay materials. IoT-based monitoring, particularly for PM1.0, underscores the ongoing need for research in developing novel IoT systems. Machine learning models, including Decision Tree, Gradient Boosting Regression, Random Forest, and Linear Regression, demonstrate potential for effective PM concentration prediction. Random Forest exhibited superior performance with Mean Absolute Error and Root Mean Squared Error values of 1.079 and 1.497, respectively. Despite lower accuracy in Linear Regression, the other three models showed proficiency in concentrations above 15 µg/m^3^, with Random Forest consistently effective. The fusion of IoT technology and machine learning provides valuable insights for assessing and managing air pollution in surface mine site, contributing to sustainable practices and human health protection.

## Data Availability

The datasets used and/or analysed during the current study available from the corresponding author on reasonable request.
